# Supplementary material for: Unveiling the Bioactive Potential of the Invasive Jellyfish Phyllorhiza punctata Through Integrative Transcriptomic and Proteomic Analyses
Source: Biomolecules. 2025 Aug 4;15(8):1121. doi: 10.3390/biom15081121 (PMC12383608; doi:10.3390/biom15081121)
Supplement: Supplementary file 1 [file biomolecules-15-01121-s001.zip › Suplementary Material/FileS3/PEPFOLD4/XP_065071194_6208/clusters.html]

 PEP-FOLD clusters


Download archive of all models  here   

|  |  |  |
| --- | --- | --- |
| # | file | sOPEP |
| # cluster 1 - Size: 16 |  |  |
|  | XP\_065071194\_6208-model1 | -9.98214 |
|  | XP\_065071194\_6208-model1.1 | -9.83643 |
|  | XP\_065071194\_6208-model1.2 | -9.02271 |
|  | XP\_065071194\_6208-model1.3 | -7.77343 |
|  | XP\_065071194\_6208-model1.4 | -7.77343 |
|  | XP\_065071194\_6208-model1.5 | -7.77343 |
|  | XP\_065071194\_6208-model1.6 | -7.77343 |
|  | XP\_065071194\_6208-model1.7 | -7.54601 |
|  | XP\_065071194\_6208-model1.8 | -7.43142 |
|  | XP\_065071194\_6208-model1.9 | -7.37567 |
|  | XP\_065071194\_6208-model1.10 | -7.33361 |
|  | XP\_065071194\_6208-model1.11 | -7.29728 |
|  | XP\_065071194\_6208-model1.12 | -7.28732 |
|  | XP\_065071194\_6208-model1.13 | -7.13908 |
|  | XP\_065071194\_6208-model1.14 | -6.96751 |
|  | XP\_065071194\_6208-model1.15 | -6.80961 |
| # cluster 2 - Size: 12 |  |  |
|  | XP\_065071194\_6208-model2 | -9.39566 |
|  | XP\_065071194\_6208-model2.1 | -8.17261 |
|  | XP\_065071194\_6208-model2.2 | -8.02375 |
|  | XP\_065071194\_6208-model2.3 | -8.0219 |
|  | XP\_065071194\_6208-model2.4 | -7.72681 |
|  | XP\_065071194\_6208-model2.5 | -7.65952 |
|  | XP\_065071194\_6208-model2.6 | -7.32092 |
|  | XP\_065071194\_6208-model2.7 | -7.26375 |
|  | XP\_065071194\_6208-model2.8 | -7.20294 |
|  | XP\_065071194\_6208-model2.9 | -7.1081 |
|  | XP\_065071194\_6208-model2.10 | -6.95093 |
|  | XP\_065071194\_6208-model2.11 | -6.72418 |
| # cluster 3 - Size: 5 |  |  |
|  | XP\_065071194\_6208-model3 | -9.27434 |
|  | XP\_065071194\_6208-model3.1 | -9.04735 |
|  | XP\_065071194\_6208-model3.2 | -8.32942 |
|  | XP\_065071194\_6208-model3.3 | -8.20311 |
|  | XP\_065071194\_6208-model3.4 | -7.63514 |
| # cluster 4 - Size: 3 |  |  |
|  | XP\_065071194\_6208-model4 | -9.13875 |
|  | XP\_065071194\_6208-model4.1 | -7.34699 |
|  | XP\_065071194\_6208-model4.2 | -6.81612 |
| # cluster 5 - Size: 7 |  |  |
|  | XP\_065071194\_6208-model5 | -9.01078 |
|  | XP\_065071194\_6208-model5.1 | -8.84007 |
|  | XP\_065071194\_6208-model5.2 | -8.49508 |
|  | XP\_065071194\_6208-model5.3 | -8.00732 |
|  | XP\_065071194\_6208-model5.4 | -8.00384 |
|  | XP\_065071194\_6208-model5.5 | -7.70435 |
|  | XP\_065071194\_6208-model5.6 | -7.00029 |
| # cluster 6 - Size: 11 |  |  |
|  | XP\_065071194\_6208-model6 | -8.90011 |
|  | XP\_065071194\_6208-model6.1 | -8.21902 |
|  | XP\_065071194\_6208-model6.2 | -8.03732 |
|  | XP\_065071194\_6208-model6.3 | -7.95802 |
|  | XP\_065071194\_6208-model6.4 | -7.94964 |
|  | XP\_065071194\_6208-model6.5 | -7.71073 |
|  | XP\_065071194\_6208-model6.6 | -7.57916 |
|  | XP\_065071194\_6208-model6.7 | -7.45435 |
|  | XP\_065071194\_6208-model6.8 | -7.37184 |
|  | XP\_065071194\_6208-model6.9 | -7.37092 |
|  | XP\_065071194\_6208-model6.10 | -7.04514 |
| # cluster 7 - Size: 3 |  |  |
|  | XP\_065071194\_6208-model7 | -8.88731 |
|  | XP\_065071194\_6208-model7.1 | -8.47294 |
|  | XP\_065071194\_6208-model7.2 | -7.29819 |
| # cluster 8 - Size: 1 |  |  |
|  | XP\_065071194\_6208-model8 | -8.74092 |
| # cluster 9 - Size: 1 |  |  |
|  | XP\_065071194\_6208-model9 | -8.25227 |
| # cluster 10 - Size: 4 |  |  |
|  | XP\_065071194\_6208-model10 | -8.24177 |
|  | XP\_065071194\_6208-model10.1 | -7.34572 |
|  | XP\_065071194\_6208-model10.2 | -7.03946 |
|  | XP\_065071194\_6208-model10.3 | -6.69406 |
| # cluster 11 - Size: 1 |  |  |
|  | XP\_065071194\_6208-model11 | -8.20888 |
| # cluster 12 - Size: 5 |  |  |
|  | XP\_065071194\_6208-model12 | -8.06523 |
|  | XP\_065071194\_6208-model12.1 | -7.9661 |
|  | XP\_065071194\_6208-model12.2 | -7.8845 |
|  | XP\_065071194\_6208-model12.3 | -7.28708 |
|  | XP\_065071194\_6208-model12.4 | -7.17727 |
| # cluster 13 - Size: 4 |  |  |
|  | XP\_065071194\_6208-model13 | -7.85358 |
|  | XP\_065071194\_6208-model13.1 | -7.82005 |
|  | XP\_065071194\_6208-model13.2 | -7.72957 |
|  | XP\_065071194\_6208-model13.3 | -7.34677 |
| # cluster 14 - Size: 1 |  |  |
|  | XP\_065071194\_6208-model14 | -7.8164 |
| # cluster 15 - Size: 5 |  |  |
|  | XP\_065071194\_6208-model15 | -7.60583 |
|  | XP\_065071194\_6208-model15.1 | -7.53721 |
|  | XP\_065071194\_6208-model15.2 | -7.47533 |
|  | XP\_065071194\_6208-model15.3 | -7.20131 |
|  | XP\_065071194\_6208-model15.4 | -6.763 |
| # cluster 16 - Size: 3 |  |  |
|  | XP\_065071194\_6208-model16 | -7.47141 |
|  | XP\_065071194\_6208-model16.1 | -7.38233 |
|  | XP\_065071194\_6208-model16.2 | -6.87291 |
| # cluster 17 - Size: 4 |  |  |
|  | XP\_065071194\_6208-model17 | -7.40957 |
|  | XP\_065071194\_6208-model17.1 | -7.15142 |
|  | XP\_065071194\_6208-model17.2 | -6.92545 |
|  | XP\_065071194\_6208-model17.3 | -6.83846 |
| # cluster 18 - Size: 1 |  |  |
|  | XP\_065071194\_6208-model18 | -7.38176 |
| # cluster 19 - Size: 4 |  |  |
|  | XP\_065071194\_6208-model19 | -7.36532 |
|  | XP\_065071194\_6208-model19.1 | -7.33517 |
|  | XP\_065071194\_6208-model19.2 | -6.93237 |
|  | XP\_065071194\_6208-model19.3 | -6.68283 |
| # cluster 20 - Size: 1 |  |  |
|  | XP\_065071194\_6208-model20 | -7.3044 |
| # cluster 21 - Size: 1 |  |  |
|  | XP\_065071194\_6208-model21 | -7.18411 |
| # cluster 22 - Size: 1 |  |  |
|  | XP\_065071194\_6208-model22 | -7.12427 |
| # cluster 23 - Size: 1 |  |  |
|  | XP\_065071194\_6208-model23 | -7.11696 |
| # cluster 24 - Size: 1 |  |  |
|  | XP\_065071194\_6208-model24 | -7.09316 |
| # cluster 25 - Size: 2 |  |  |
|  | XP\_065071194\_6208-model25 | -6.90946 |
|  | XP\_065071194\_6208-model25.1 | -6.79348 |
| # cluster 26 - Size: 1 |  |  |
|  | XP\_065071194\_6208-model26 | -6.807 |
| # cluster 27 - Size: 1 |  |  |
|  | XP\_065071194\_6208-model27 | -6.69667 |
|  |  |  |
